# MicroRNA in exosomes isolated directly from the liver circulation in patients with metastatic uveal melanoma

**DOI:** 10.1186/1471-2407-14-962

**Published:** 2014-12-16

**Authors:** Maria Eldh, Roger Olofsson Bagge, Cecilia Lässer, Joar Svanvik, Margareta Sjöstrand, Jan Mattsson, Per Lindnér, Dong-Sic Choi, Yong Song Gho, Jan Lötvall

**Affiliations:** Krefting Research Centre, Department of Internal Medicine and Clinical Nutrition, Sahlgrenska Academy, University of Gothenburg, Gothenburg, Sweden; Department of Surgery, Institute of Clinical Sciences, Sahlgrenska Academy at University of Gothenburg, Sahlgrenska University Hospital, Gothenburg, Sweden; Transplant Institute, Sahlgrenska Academy at University of Gothenburg, Sahlgrenska University Hospital, Gothenburg, Sweden; Division of Molecular and Life Sciences, Department of Life Science, Pohang University of Science and Technology (POSTECH), Pohang, South Korea

**Keywords:** Exosomes, Extracellular vesicles, Biomarker, microRNA, Uveal melanoma, Liver perfusion

## Abstract

**Background:**

Uveal melanoma is a tumour arising from melanocytes of the eye, and 30 per cent of these patients develop liver metastases. Exosomes are small RNA containing nano-vesicles released by most cells, including malignant melanoma cells. This clinical translational study included patients undergoing isolated hepatic perfusion (IHP) for metastatic uveal melanoma, from whom exosomes were isolated directly from liver perfusates. The objective was to determine whether exosomes are present in the liver circulation, and to ascertain whether these may originate from melanoma cells.

**Methods:**

Exosomes were isolated from the liver perfusate of twelve patients with liver metastases from uveal melanoma undergoing IHP. Exosomes were visualised by electron microscopy, and characterised by flow cytometry, Western blot and real-time PCR. Furthermore, the concentration of peripheral blood exosomes were measured and compared to healthy controls.

**Results:**

The liver perfusate contained Melan-A positive and RNA containing exosomes, with similar miRNA profiles among patients, but dissimilar miRNA compared to exosomes isolated from tumor cell cultures. Patients with metastatic uveal melanoma had a higher concentration of exosomes in their peripheral venous blood compared to healthy controls.

**Conclusions:**

Melanoma exosomes are released into the liver circulation in metastatic uveal melanoma, and is associated with higher concentrations of exosomes in the systemic circulation. The exosomes isolated directly from liver circulation contain miRNA clusters that are different from exosomes from other cellular sources.

**Electronic supplementary material:**

The online version of this article (doi:10.1186/1471-2407-14-962) contains supplementary material, which is available to authorized users.

## Background

In the 1980’s, nano-sized extracellular vesicles called exosomes were identified and characterised [1–4]. Exosomes are considered to be formed through the endosomal pathway and are released by fusion of multivesicular bodies with the plasma membrane [5]. These vesicles are released by virtually all cells examined, including epithelial cells [6], immune cells [7–9] and tumour cells [10]. Exosomes were first thought to function as the cellular “garbage bin”, eradicating unwanted proteins [1, 3], but in the 1990’s, exosomes were shown to have an immunoregulatory function [7]. Exosomes are also present in many human body fluids, such as malignant effusions [11], human blood plasma [12], urine [13] and breast milk [14]. Extracellular vesicles, including exosomes, harbour extracellular RNA with potentially multiple functions in cell-to-cell communication [15–17], and are being tested as markers for diagnosis and prognosis of different diseases [18, 19].

MicroRNAs (miRNAs) are a class of small (~22 nucleotides) endogenously expressed, non-coding RNA molecules, which act as gene regulators. They regulate the gene expression by repressing it at a post-transcriptional level by binding to complementary sequences usually at the 3’ untranslated regions of target mRNAs [20, 21]. These small regulators of gene expression are involved in diverse cellular processes, including cell proliferation, cell death, angiogenesis and cancer development [22–24].

Uveal melanoma is a malignant tumour that arises in the melanocytes of the eye, and tumours originating from either the ciliary body or the choroid are collectively referred to as posterior tract uveal melanomas [25]. Despite successful control of the primary tumour, approximately one third of the patients will develop metastases, predominantly liver metastases (90%) [26]. The prognosis is poor with a median survival of about 6 months, and no systemic treatment has been shown to improve survival [26]. Different regional treatment strategies including liver resection [27], chemo-embolisation [28] and isolated hepatic perfusion (IHP) have been explored. During IHP the liver is completely isolated from the systemic circulation, allowing a high concentration of chemotherapeutics to be perfused through the liver with minimal exposure to the systemic circulation [29].

The aim of this study was to determine whether exosomes of uveal melanoma origin can be obtained directly from the isolated local liver circulation in patients with liver metastases undergoing IHP. Plasma from the liver perfusates of twelve patients undergoing IHP were collected prior to the local perfusion chemotherapy. Exosomes were visualised and characterised using electron microscopy, flow cytometry and Western blot, with exosomal RNA profiles determined by capillary electrophoresis and microRNAs with real-time PCR.

## Methods

### Patients

During the period of November 2010 to October 2012, twelve patients with isolated liver metastases from uveal melanoma underwent treatment with IHP. Inclusion criteria included less than 50% of the liver replaced by tumour and no extra hepatic tumour manifestations. Patient characteristics are summarised in Table 1. The study was approved by the Regional Ethical Review Board at the University of Gothenburg (Ethical permit id 096-12) and all participants provided a written informed consent.

**Table 1:** **Patient characteristics**

	***Primary tumour***		***Liver metastases***		***Clinical outcome***		
***No.***	***Type***	***Treatment***	***Tumour burden***	***Largest diameter***	***Response***	***Status***	***Laboratory characterisation of exosomes***
1	Choroidal	I-125	10-24%	15 mm	PR	Alive 29 mo	RNA, FACS, PCR
2	Choroidal	En	25-50%	25 mm	PR	Dead 25 mo	RNA
3	Choroidal	I-125 + En	10-24%	45 mm	PR	Dead 17 mo	RNA
4	Choroidal	En	<10%	20 mm	PR	Alive 17 mo	EM
5	Choroidal	En	<10%	55 mm	PR	Dead 14 mo	EC, RNA, PCR
6	Choroidal	En	10-24%	35 mm	PR	Alive 13 mo	EC, RNA, FACS
7	Choroidal	I-125	<10%	23 mm	SD	Alive 13 mo	EC, RNA, PCR
8	Choroidal	Ru-106	25-50%	100 mm	PD	Alive 11 mo	EC, RNA, PCR, FACS
9	Choroidal	En	<10%	40 mm	PR	Alive 10 mo	EC, RNA, PCR
10	Choroidal	Ru-106 + En	<10%	30 mm	SD	Alive 7 mo	EC
11	Choroidal	En	<10%	15 mm	SD	Alive 6 mo	EC
12	Choroidal	En	10-24%	25 mm	PR	Alive 5 mo	EC

I-125 = Iodine-125 brachytherapy; Ru-106 = Ruthenium-106 brachytherapy; En = Enucleation; PR = Partial response; SD = Stable disease; PD = Progressive disease; EC = Exosome concentration (peripheral blood); RNA = RNA concentration (liver perfusate); FACS = Flow cytometry (liver perfusate); PCR = Exosomal miRNA PCR array (liver perfusate); EM = Electron microscopy (liver perfusate).

### Isolated liver perfusion

**Figure 1:**
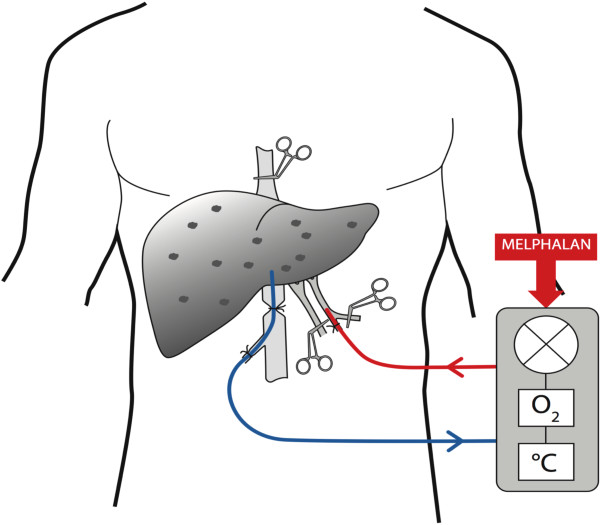
**Schematic representation of isolated hepatic perfusion.** The figure shows the setup for isolated hepatic perfusion (IHP), where the liver is isolated by clamping and cannulation of the hepatic artery and the inferior caval vein. The liver is then connected to an oxygenated extracorporeal circuit and the portal vein is clamped. Melphalan is then administered into the perfusion circuit.

The IHP technique was performed as described previously [29]. In brief, the liver was completely mobilised and isolated by the clamping and cannulation of the hepatic artery and the inferior caval vein. The cannulas were connected to an oxygenated extracorporeal circuit and the portal vein was clamped (Figure 1). The liver was perfused with a priming solution consisting of one unit of erythrocytes, 100 ml of human albumin (50 mg/ml; Baxter, Deerfield, IL, USA), 100 ml of buffer (Tribonat, Fresenius Kabi, Uppsala, Sweden), 2500 units of heparin (LEO Pharmaceutical Products, Copenhagen, Denmark) and 250 ml of Ringer’s solution (Ringer Acetat, Baxter, Deerfield, IL, USA). Thereafter, melphalan (1 mg/kg body weight) was administered into the perfusion system. The temperature was held at 40°C with a total perfusion time of 60 minutes. After perfusion, the liver was washed with 1000 ml of Ringer’s solution (Baxter) and one unit of erythrocytes was added.

### Sample collection and exosome isolation

After 10 minutes of perfusion with priming solution, before melphalan was added, approximately 200 ml of the liver perfusate was collected for this study. In addition to the liver perfusate, peripheral blood samples were taken prior to the IHP procedure.

Plasma was obtained from both the liver perfusate and peripheral heparinised blood by centrifugation at 400 × g for 10 minutes, followed by a second centrifugation at 1 880 × g for 10 minutes. The purified blood plasma was then centrifuged at 29 500 × g for 20 minutes to remove remaining cells and cell debris. The supernatant was filtered through 0.2 μm filters to remove particles larger than 200 nm. Exosomes were pelleted from the purified blood plasma by ultracentrifugation at 120 000 × g for 90 minutes (Ti70 rotor, Beckman Coulter, Brea, CA, USA).

### Cell culture and exosome isolation

The human malignant melanoma cell line A375 (ATCC, Manassas, VA, USA), was cultured in DMEM medium. The human malignant melanoma cell line, MML-1 (CLS, Eppelheim, Germany), the human breast cancer cell line, HTB-133 (ATCC), and the human lung carcinoma cell line, HTB-177 (ATCC), were all cultured in RPMI-1640. The human mast cell line, HMC-1.2 (Dr. Joseph Butterfield, Mayo Clinic, Rochester, MN, USA), was cultured in IMDM. The complete growth media for all cells contained 10% fetal bovine serum (FBS), 100 units/ml penicillin, 100 μg/ml streptomycin and 2 mM L-glutamine (all from Sigma-Aldrich, St Louis, MO, USA). In addition, the A375 cell line complete growth medium also contained 1% Non-Essential Amino Acids (PAA laboratories, Pasching, Austria), the HTB-133 complete growth medium also contained 0.2 units/ml insulin, and the HMC-1.2 complete growth medium also contained 1.2 mM alpha-thioglycerol (both from Sigma-Aldrich). Prior to use, the FBS was ultracentrifuged at 120 000 × g overnight to eliminate serum exosomes. All cells were cultured at 37°C and 5% CO_2_.

Exosomes were purified from the cell cultures by centrifuging the cells at 300 × g for 10 minutes, followed by 16 500 × g for 20 minutes, to remove remaining cells and cell debris. The supernatant was then filtered through 0.2 μm filters and exosomes were finally pelleted by ultracentrifugation at 120 000 × g for 70 minutes (Ti45 rotor, Beckman Coulter).

### Flow cytometry analysis

For immunoisolation of exosomes, 4 μm-diameter aldehyde/sulphate latex beads (Interfacial Dynamics, Portland, OR, USA) were incubated with purified anti-CD63 antibody (BD Biosciences, Erembodegem, Belgium) at room temperature (RT) overnight with gentle agitation, as previously described [30]. For flow cytometry analysis, isolated plasma exosomes were resuspended in PBS and 30 μg of exosomes were incubated with 1.5 × 10^5^ anti-CD63 beads for 15 minutes at RT. PBS was then added up to 300 μl and the beads incubated at 4°C overnight with gentle agitation. The reaction was stopped by incubation of the exosome-bead complexes in 100 mM glycine for 30 minutes. The exosome-bead complexes were washed twice in FACS buffer (3% exosome depleted FBS in PBS). The exosome-bead complexes were then incubated in human IgG (Sigma-Aldrich) at 4°C for 15 minutes, followed by incubation with PE-conjugated anti-CD9 (clone M-L13), anti-CD63 (clone H5C6), anti-CD81 (clone JS-81) antibodies or isotype control (all antibodies were from BD Biosciences) for 40 minutes at RT with gentle agitation. The complexes were washed twice, resuspended in 300 μl FACS buffer and analysed by flow cytometry using a FACSAria (BD Biosciences, San Jose, CA, USA), and the FlowJo Software (Tri Star Inc, Ashland, OR, USA).

### Electron microscopy

Formvar/carbon-coated nickel grids (Ted Pella Inc, Redding, CA, USA) were UV-treated for 5 minutes before 20 μg of exosomes, re-suspended in PBS, were loaded and incubated for 10 minutes. The exosomes were then fixed in 2% paraformaldehyde and washed with PBS. The exosomes were immunostained with anti-CD63 antibody (BD Bioscience) or isotype control (Sigma-Aldrich), followed by staining with a 10 nm gold-labelled secondary antibody (Sigma-Aldrich). The exosomes were then fixed in 2.5% glutaraldehyde, washed in ddH_2_O and contrasted in 2% uranyl acetate. The preparations were examined using a LEO 912AB Omega electron microscope (Carl Zeiss NTS, Jena, Germany).

### Western blot

Purified exosomes were treated with lysis buffer (20 mM Tris HCL/1% SDS) and subjected to sonication and vortexing, with protein concentration determined using a BCA protein assay kit (Thermo Scientific Pierce, Rockford, IL, USA) according to manufacturer’s recommendations. The exosomal proteins (50 μg) were run on a Mini-Protean TGX precast gel (Any kD, Bio-Rad Laboratories, Hercules, CA, USA) and blotted to Trans-Blot Mini PVDF membranes using the Trans-Blot® Turbo™ Transfer system (Bio-Rad). Blots were processed using the Snap i.d. protein detection system (Millipore, Billerica, MA, USA) according to manufacturer’s recommendations. Melan-A was detected using an anti-Melan-A antibody (1:250; FL-118, sc-28871, Santa Cruz Biotechnology, Santa Cruz, CA, USA) together with a donkey-anti-rabbit secondary antibody (1:10000; NA9340, GE Healthcare, Little Chalfont, UK) and visualised by enhanced chemiluminescence (GE Healthcare), the VersaDoc™ 4000 MP Imaging System and Quantity One® software (both from Bio-Rad).

### Total RNA analysis

Total RNA was extracted from exosomes using the miRCURY™ RNA Isolation Kit (Exiqon, Vedbaek, Denmark) according to the manufacturer’s protocol. The RNA yield and size distribution pattern of exosomal total RNA was analysed using capillary electrophoresis (Agilent 2100 Bioanalyzer, Agilent Technologies, Foster City, CA, USA) with the total RNA 6000 Nano and 6000 Pico Kit, according to manufacturer’s protocol.

### MicroRNA analysis

Purified liver perfusion exosomes from five patients were analysed using a panel of 88 brain cancer-related miRNA using RT^2^ miRNA PCR arrays (MAH-108A, Qiagen, Germantown, MD, USA). As controls, exosomes purified from multiple cell lines were used; A375, MML-1, HTB-133, HTB-177 and HMC-1.2. Total RNA was digested with Turbo DNase (Turbo DNase Free kit, Ambion, Carlsbad, CA, U.S), reverse transcribed using the RT^2^ miRNA First Strand Kit (Qiagen), and subjected to real-time PCR in a Bio-Rad CFX96 Real-Time PCR Detection System (Bio-Rad), using RT^2^ SYBR® Green qPCR Mastermix (Qiagen). The cDNA was amplified for 10 minutes at 95°C and 40 cycles x [95°C, 15 sec; 60°C, 30 sec; 72°C, 30 sec] and finalised by a dissociation curve 65°C to 95°C at 2°C/minute. The results were analysed using the CFX Manager™ software (Bio-Rad).

MiRNA expression levels were measured by the threshold cycle (Ct). Samples with a Ct value over 35 and samples showing more than one melt peak, with a ratio less than 80% between the major peak and the secondary peak, were excluded from the analysis. As no reference genes are available for exosomes, the miRNA expression levels by the Ct value could not be compared between samples. Thus, the miRNA of each sample were ranked according to their Ct values and the rank lists were compared between the different patients and cell lines (Additional file 1: Table S1).

The miRNAs were then clustered according to rank, rather than Ct value, using Cluster 3.0 [31]. Clustering analyses were performed with a hierarchical method using average linkage and Euclidean distance metric to illustrate relationships between the different patients and cell lines. A heat map of the clustering data was visualised using Microsoft Excel.

Enriched KEGG pathway analyses for miRNAs was conducted via DIANA-miRPath v.2.0 software based on predicted targets by DIANA-microT-CDS [32]. Targets of miRNAs with a score of more than 0.8 were selected. Only KEGG pathways including at least five genes and displaying an enrichment p-value of less than 0.05 were considered significant.

### Statistical analysis

Where appropriate, data are expressed as mean ± SD. Statistical analysis was performed by using the Mann-Whitney non-parametric test. A p-value less than 0.05 was accepted as statistically significant.

## Results

### Clinical characteristics

All twelve patients included underwent IHP without complications. Response evaluation with CT or MRI scans three months after IHP showed a partial response in eight patients, three patients with stable disease and one patient that showed progression of disease (all according to RECIST: Response Evaluation Criteria in Solid Tumors criteria) [33]. Three patients died 25, 17 and 14 months after IHP, while the other nine patients are still alive (Table 1).

### Liver perfusates contains exosomes

**Figure 2:**
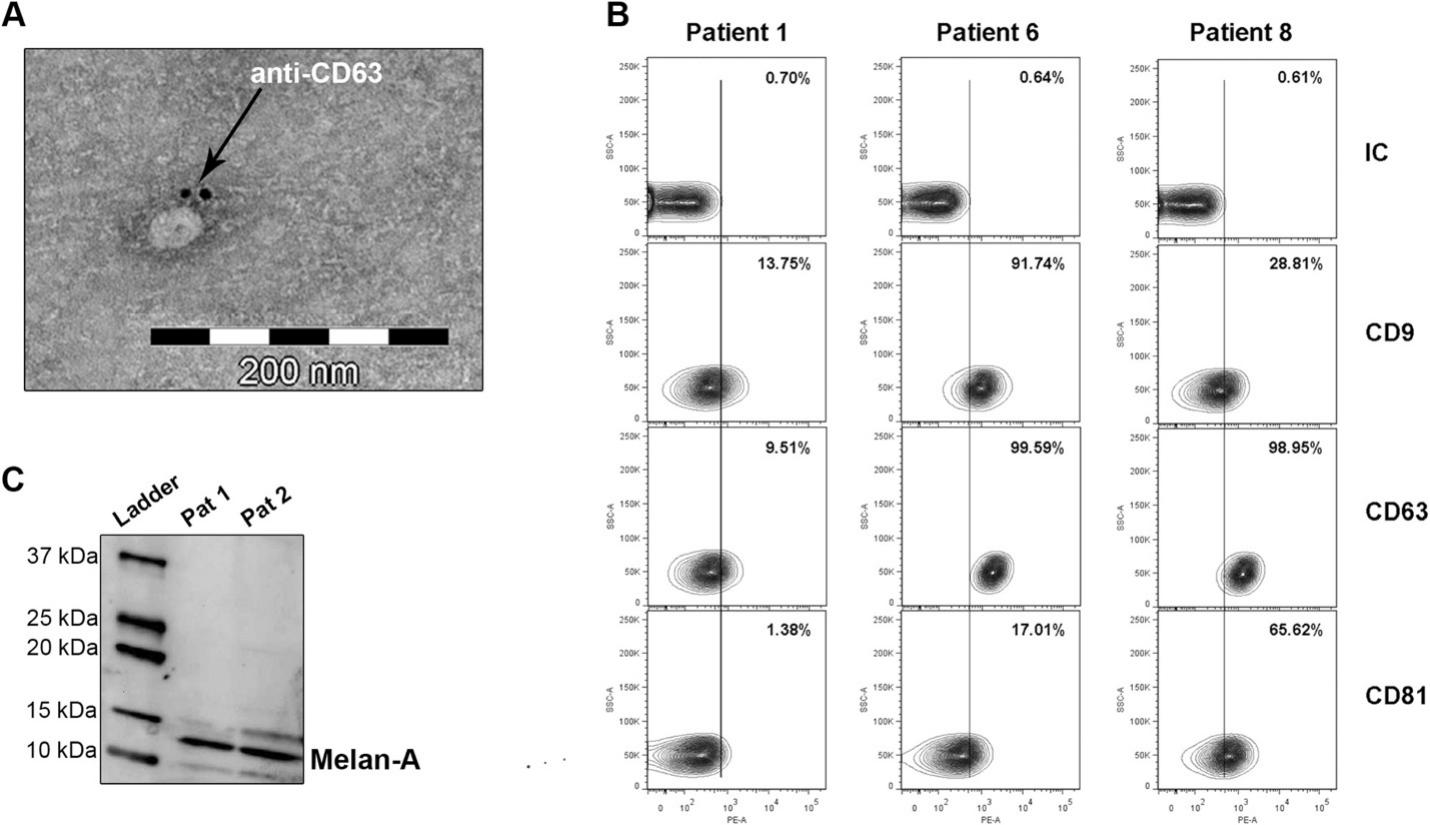
**Liver perfusate contains melanoma-specific exosomes. (A)** A representative electron microscopy image of an exosome derived from the liver perfusate of a patient (patient 4) with metastatic uveal melanoma. The image shows a small vesicle, approximately 50 nm in diameter, and immune-gold labelled with anti-CD63. **(B)** Flow cytometry analysis showing exosomes coupled to CD63 coated latex beads and immunostained with antibodies against the tetraspanins CD9, CD63, CD81 and isotype control. All three patients were shown to be positive for the three markers, with variations between the patients. **(C)** A representative Western blot analysis showing liver perfusate exosomes from two patients positive for the melanoma-specific marker Melan-A. Abbreviations: IC = Isotype control, Pat = Patient.

Exosomes isolated from liver perfusate from one patient showed a typical spherical shape in electron microscopy, with a size of about 50 nm and positive for CD63 using immuno-gold staining (Figure 2A). Further analysis with flow cytometry, using anti-CD63 coated latex beads, showed the presence of CD9, CD63 and CD81 on the exosomal surface, with expression differences between the patients (Figure 2B). Additionally, Western blot analyses showed exosomes positive for the melanoma-specific marker Melan-A, indicating that the liver perfusate exosomes are of tumour origin (Figure 2C).

### Patients have more circulating exosomes compared to healthy controls

**Figure 3:**
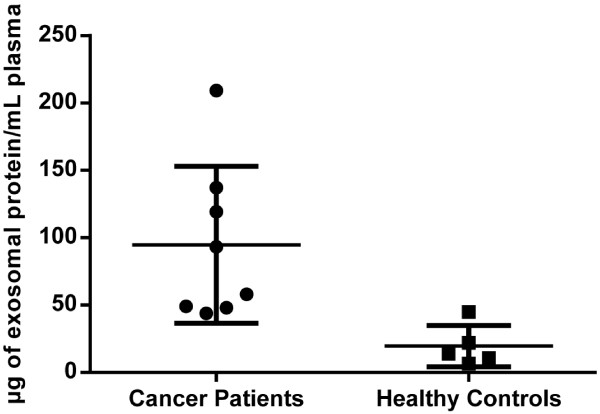
**Patients have higher levels of circulating exosomes compared to healthy controls.** Exosomes isolated from peripheral blood plasma of patients (n = 8) with metastatic uveal melanoma were shown to contain significantly more exosomes compared to healthy controls (n = 5), according to the total amount of protein (μg) in circulating exosomes per ml of plasma analysed (** = p-value 0.003).

To determine whether the concentration of exosomes differ in the systemic circulation in patients compared to healthy controls, exosomes were isolated from peripheral blood before IHP. The exosome concentration, measured as total exosomal protein, differed significantly between the peripheral blood of healthy controls and patients (median 13.8 [range 6-45] vs. 75.6 [range 44-209] μg/ml plasma, p = 0.003) (Figure 3).

### RNA profiles and miRNA analyses of liver perfusate exosomes

**Figure 4:**
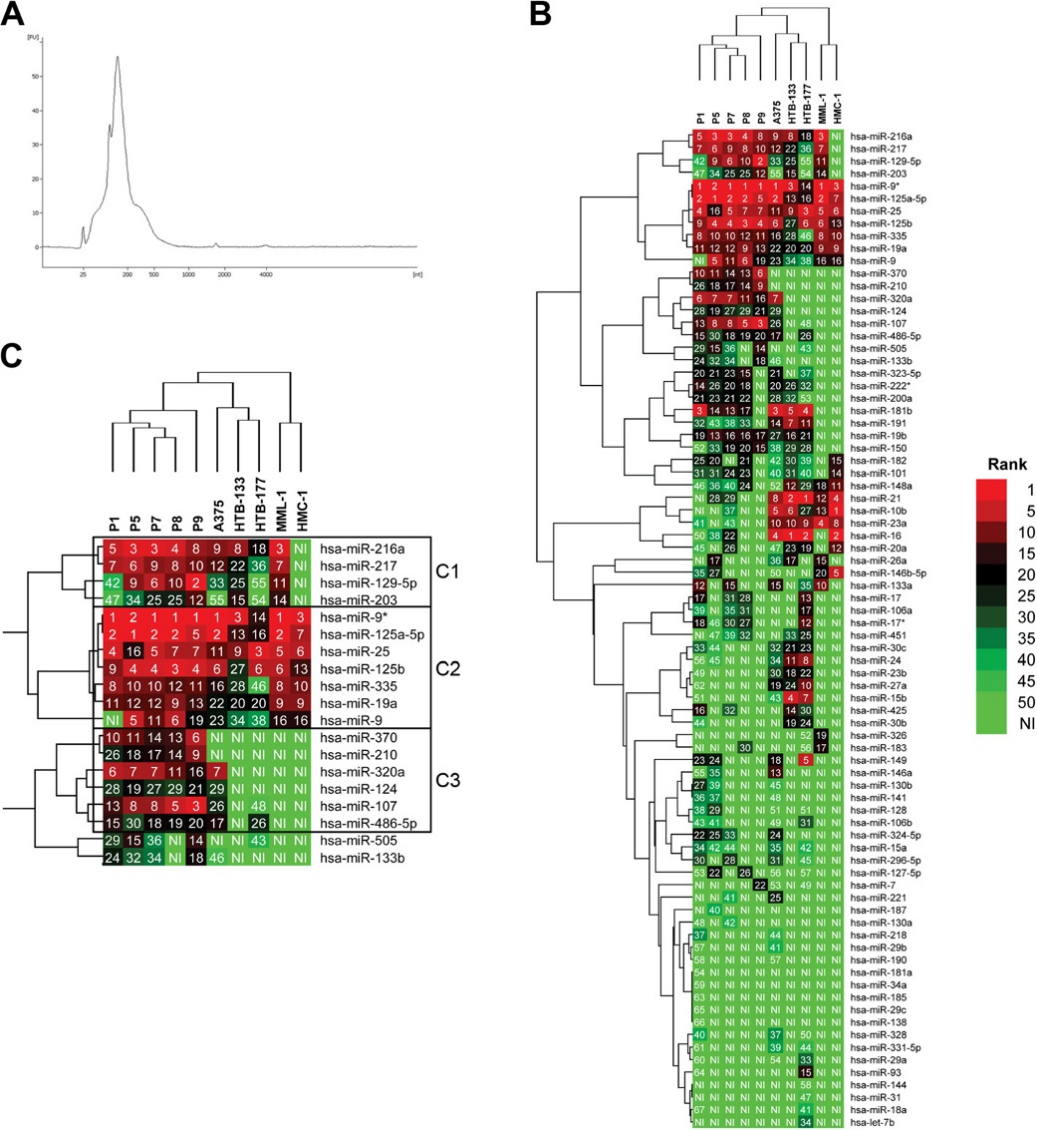
**RNA profile of liver perfusate exosomes. (A)** A representative Bioanalyzer electropherogram of liver perfusate exosomes, showing a classical exosomal RNA profile, lacking the ribosomal 18S and 28S subunits, and enriched in lower molecular weight RNAs. **(B)** Cluster analysis of the miRNAs found in exosomes from patients and cell lines, showing a clear similarity between the patients. **(C)** A close-up of a specific portion of the cluster analysis, highlighting three miRNA clusters. Cluster 3 shows miRNAs that are more associated with patients than control cell lines. Abbreviations: P1-9 = Patient 1 to 9, C1-3 = Cluster 1 to 3, NI = Not identified.

The RNA profile of liver perfusate exosomes were analysed by capillary electrophoresis, which showed a typical exosomal RNA profile, lacking the 18S and 28S ribosomal subunits (Figure 4A). The electrophoresis profile included RNA in the size of miRNA and to verify that the exosomes contain miRNA, the exosomes were further analysed by real-time PCR. The most abundant exosomal miRNAs correlated well between patients (Additional file 1: Table S1).

As appropriate *in vivo* controls are unavailable, we chose to compare the miRNA profiles with two malignant melanoma cell lines (A375 and MML-1), one breast cancer cell line (HTB-133), one lung cancer cell line (HTB-177) and one human mast cell line (HMC-1). Comparison of the exosomal miRNA profiles of patients and cell lines using cluster analysis revealed a clear relationship between the patient samples. Interestingly, the two melanoma cell lines did not cluster together, but did form a cluster together with the other cell lines (Figure 4B).

The three top miRNA clusters were analysed in more detail. Cluster 1 consisted of four miRNAs (miR-216a, miR217, miR129-5p and miR-203) that were expressed almost equally in patients and the lung (HTB-177), breast (HTB-133) and melanoma cell lines (A375 and MML-1), but not in the human mast cell line HMC-1 (Figure 4C). KEGG pathway analysis showed that metabolism and signalling pathways, including “glycosaminoglycan biosynthesis” and “TGF-beta signalling” (Additional file 2: Table S2), were among the most common pathways.

Cluster 2 consisted of seven miRNAs (miR-9*, miR125a-5p, miR-25, miR-125b, miR-335, miR-19a and miR-9) that were also expressed almost equally in patients and the five tested cell lines (Figure 4C). KEGG pathway analysis showed that biosynthesis and metabolism pathways, such as “glycosaminoglycan biosynthesis”, “mucin type-O-glycan biosynthesis” and “biotin metabolism” (Additional file 3: Table S3), were among the most common pathways.

Cluster 3 consisted of six miRNAs (miR-370, miR-210, miR-320a, miR-124, miR-107 and miR-486-5p) that were primarily present in patients, and were also measurable in the A375 melanoma cell line, but not present in the other four cell lines (Figure 4C). KEGG pathway analysis showed that “melanoma”, together with “glioma”, “hedgehog signalling” and “prostate cancer” were the most significant pathways with p-values less than 0.01 (Table 2).

**Table 2:** **Enriched KEGG pathways of predicted targets for the six miRNAs (miR-370, miR-210, miR-320a, miR-124, miR-107 and miR-486-5p) in cluster 3**

KEGG pathway	P value	Number of genes	Genes
Hedgehog signalling	0.009	14	BMP2, BMP6, BTRC, CSNK1A1, CSNK1E, CSNK1G3, GLI3, IHH, LRP2, RAB23, SMO, SUFU, WNT16, WNT3A
Glioma	0.010	11	AKT3, CDK4, CDK6, E2F3, IGF1, IGF1R, MAPK1, PIK3CA, PIK3R1, PTEN, SOS2
Melanoma	0.010	15	AKT3, CDK4, CDK6, E2F3, FGF19, FGF2, FGF7, IGF1, IGF1R, MAPK1, PDGFC, PDGFD, PIK3CA, PIK3R1, PTEN
Prostate cancer	0.010	18	AKT3, CCNE1, CREB3, CREB3L2, CREB5, CTNNB1, E2F3, FOXO1A, IGF1, IGF1R, LEF1, MAPK1, PDGFC, PDGFD, PIK3CA, PIK3R1, PTEN, SOS2
Focal adhesion	0.014	30	AC128683.3, AKT3, ARHGAP5, COL4A1, CRKL, CTNNB1, IGF1, IGF1R, ITGA1, ITGA2, ITGA4, ITGB8, LAMC1, MAPK1, MAPK8, MYL12A, PDGFC, PDGFD, PIK3CA, PIK3R1, PPP1R12A, PTEN, RAC1, RAP1A, RAPGEF1, SOS2, VAV3, VCL, XIAP, ZYX
Pathways in cancer	0.014	43	AKT3, ARNT, AXIN2, BMP2, CBL, CCNE1, CDK4, CDK6, COL4A1, CRKL, CTNNB1, DVL1, E2F3, EVI1, FGF19, FGF2, FGF7, FOXO1A, GLI3, IGF1, IGF1R, ITGA2, KITLG, LAMC1, LEF1, MAPK1, MAPK8, PIK3CA, PIK3R1, PTEN, RAC1, RASSF5, RET, RUNX1T1, SMO, SOS2, STK4, SUFU, TPM3, VHL, WNT16, WNT3A, XIAP
Circadian rhythm - mammal	0.015	6	ASH1L, EHHADH, MLL5, SETD1B, SUV420H1, WHSC1
mTOR signalling	0.017	12	AC026713.5, AKT3, CAB39, EIF4B, IGF1, MAPK1, PIK3CA, PIK3R1, PRKAA2, RPS6KA3, TSC1, ULK1
Adipocytokine signalling	0.038	10	ADIPOR1, AKT3, JAK2, LEPR, MAPK8, PPARGC1A, PRKAA2, PRKAB2, PRKAG2, PRKAG3
Lysine degradation	0.040	6	ASH1L, EHHADH, MLL5, SETD1B, SUV420H1, WHSC1
Insulin signalling	0.041	21	AKT3, CBL, CRKL, FASN, FOXO1A, INSR, MAPK1, MAPK8, PDE3B, PHKA1, PIK3CA, PIK3R1, PPARGC1A, PRKAA2, PRKAB2, PRKAG2, PRKAG3, RAPGEF1, SKIP, SOS2, TSC1
Endometrial cancer	0.049	9	AKT3, AXIN2, CTNNB1, LEF1, MAPK1, PIK3CA, PIK3R1, PTEN, SOS2
Cytokine-cytokine receptor interaction	0.049	18	ACVR2A, ACVR2B, BMP2, BMPR1A, BMPR2, CCL21, CCR7, CCR8, CXCR5, IL1RAP, IL23R, IL4R, IL6ST, IL9R, KITLG, LEPR, LIFR, PDGFC

## Discussion

Patients with different malignant diseases have detectable tumour-derived exosomes in their peripheral blood [18, 19, 34]. Patients with uveal melanoma and liver metastases have a poor prognosis, with very few treatment options. IHP is a unique surgical technique with complete isolation of the liver, enabling high concentrations of chemotherapeutics to be delivered to the liver with minimal systemic toxicity. This method also offers a unique possibility to study exosomes from the liver of patients with uveal melanoma liver metastases. During liver perfusion, before adding any chemotherapeutic drug, samples were collected with the hypothesis that it would be possible to collect a high concentration of tumour-specific exosomes from the liver perfusate. The results show, using electron microscopy and flow cytometry, that the liver perfusate indeed contain significant quantities of exosomes (Figure 2A and B). Furthermore, exosomes stained positive for the melanoma-specific marker Melan-A, suggesting a tumour origin for at least a portion of the exosomes investigated (Figure 2C). Furthermore, we show that these patients have higher levels of circulating exosomes in the systemic circulation compared to healthy controls. Importantly, miRNA profiling suggests that exosomes from liver perfusates may have different miRNAs compared to exosomes released from other types of malignant cells.

Various studies have shown that exosomes may promote tumour vasculogenesis, invasion and metastasis [34–36]. In addition, the content of exosomes has been suggested to have prognostic value in both melanoma and other cancers [18, 19, 34]. Peinado and co-workers showed that specific melanoma signatures could be seen in circulating exosomes from subjects with advanced melanoma [34]. Interestingly, in addition to the specific profile, they also showed that the amount of protein per exosome increased with disease. In this study we confirm that patients with metastatic uveal melanoma have a significantly higher level of exosomes in their peripheral blood plasma compared to healthy controls (Figure 3), in agreement with the results of Peinado and co-workers, supporting the potential role of exosomes as possible diagnostic markers.Liver perfusate-derived exosomes were shown to contain RNA with a typical exosomal profile, including miRNA sized RNA (Figure 4A), with the presence of miRNA verified by real-time PCR. The lack of a commercially available uveal melanoma miRNA PCR array panel necessitated the use of a non-specific miRNA array, which contained miRNAs known to be associated with uveal melanoma. Such developments could be helpful for future biomarker studies in the uveal melanoma field.

The miRNA profiles were shown to be similar among patients with metastatic uveal melanoma (Figure 4B). Attempts to compare these miRNAs with RNA obtained from peripheral blood of the healthy controls failed, as the amount of isolated RNA containing exosomes was insufficient for real-time PCR analyses. This is in accordance with previous studies showing that healthy controls have substantially lower levels of circulating exosomes compared to cancer patients [19]. Consequently, five different cell lines were used as controls to determine whether the pattern observed among the patients was associated with melanoma.

Interestingly, clustering of the miRNA profiles showed a strong similarity among the patients, but not directly between the patients and the different malignant cell lines. The analysis revealed multiple miRNA clusters, with cluster 1 and 2 (Figure 4C) being similar among patients and four of the five cell lines. These two clusters primarily included pathways involved in signalling and metabolism (Additional file 2: Table S2 and Additional file 3: Table S3). This may suggest that the function of these miRNAs are associated with general biology, rather than with specific melanoma processes. In cluster 3, a number of miRNAs (miR-370, miR-210, miR-320a, miR-124, miR-107 and miR-486-5p) were identified that could not be detected in exosomes from most of the cell lines. These miRNAs could reflect an up regulation in the exosome-producing tumour cell, as it has previously been suggested that the RNA content in tumour exosomes can reflect the RNA in the tumour cells [18]. However, it was recently demonstrated that cancer cells can release tumour-suppressive miRNA via exosomes to promote a more metastatic phenotype [37], which might instead suggest that these miRNA are disposed by the tumour cells to become more aggressive. Importantly, the KEGG pathway analysis showed that the miRNAs in cluster 3 were highly associated with the pathway of “melanoma”, again arguing that the analysis is relevant for the disease studied and might suggest that the miRNA are reflecting the tumour cells and is not released to dispose unwanted tumour-suppressive miRNA. Furthermore, the cluster analysis showed a strong similarity between the five patients, but also some similarity with one of the tested melanoma cell line, A375. This argues that some of the exosomes isolated from the liver circulation, are produced by the liver metastases.

The role of each specific miRNA in the biology of uveal melanoma metastases is complicated to determine, and beyond the scope of this study. Nevertheless, it is still possible to consider several putative roles of some of the most prominent miRNAs found in this, and previous, studies. For example, both miR-182 and miR-34a were shown to be low or absent in our patient material. MiR-182 functions as a tumour suppressor *in vitro* in uveal melanoma by inhibiting cell proliferation, migration and invasion, by down-regulation of the microphthalmia-associated transcription factor and the proto-oncogene, c-Met. Furthermore, miR-182 has been shown to suppress the anti-apoptotic gene, BCL2, and to reduce tumour cell growth *in vivo* [38]. Additionally, miR-34a acts as a tumour suppressor in both uveal melanoma [39] and glioblastoma [40] by the down-regulation of c-Met. The down-regulation of these specific miRNAs in uveal melanoma correlates with our results, thus indicating that the miRNA profiles seen in the patients may be associated with uveal melanoma. Another study, comparing seven different miRNAs between primary uveal melanoma and control specimens, found that miR-20a, miR-106a, miR-34a and miR-21 were significantly up-regulated in primary tumours. These miRNAs were low or absent in our patient material, with the exception of miR-21, which was ranked relatively high in some of the cell lines [41]. A direct comparison between these results may not be possible as our results are based on exosomal miRNA from liver metastases rather than from primary tumours.

An important limitation of this study is the low number of patients included, as well as the relatively low concentration of exosomes in the sampled plasma. Both of these drawbacks mean that the number of analyses per patient and exosomes sample is limited, and that extensive characterisation with current technologies cannot be performed in every single patient. On the other hand, the liver perfusates are exceptionally unique, and may be even more relevant than peripheral blood exosomes samples, as they come from the circulation that is most likely to be enriched in metastasis-derived exosomes. Further, the IHP procedure is performed in very few centers globally, and no reports about exosomes in uveal melanoma have been published.

## Conclusions

Overall, these findings show that exosomes can be isolated directly from the local blood circulation of an organ with metastases, in this case the liver. The presence of Melan-A and melanoma-associated microRNAs argue that the isolated exosomes are indeed originating from the melanoma metastases, although some liver cell-derived exosomes may have been co-isolated with the melanoma-derived exosomes. Future studies in larger patient populations will help determine whether exosomes, and their microRNA and/or protein cargo, can help diagnosing the progression of uveal melanoma.

## Electronic supplementary material

Additional file 1: Table S1: MiRNA profile of purified liver perfusion exosomes (n=5), from patients with uveal melanoma, and five cell lines used as controls, were analysed for the presence of a panel of 88 cancer-related miRNA using RT2 miRNA PCR. The table shows all miRNA that passed quality control; Samples with a Ct value over 35, and samples showing more than one melt peak, with a ratio less than 80% between the major peak and the secondary peak, were excluded from the analysis. As no reference genes are available, miRNA were ranked according to their Ct values for each sample. (PDF 95 KB)

Additional file 2: Table S2: Enriched KEGG pathways of predicted targets for the four miRNAs (miR-216a, miR217, miR129-5p and miR-203) in cluster 1. (PDF 26 KB)

Additional file 3: Table S3: Enriched KEGG pathways of predicted targets for the seven miRNAs (miR-9*, miR125a-5p, miR-25, miR-125b, miR-335, miR-19a and miR-9) in cluster 2. (PDF 17 KB)
