# Supplementary material for: MicroRNA in exosomes isolated directly from the liver circulation in patients with metastatic uveal melanoma
Source: BMC Cancer. 2014 Dec 16;14:962. doi: 10.1186/1471-2407-14-962 (PMC4320618; doi:10.1186/1471-2407-14-962)
Supplement: Supplementary file 1 — Additional file 1: Table S1: MiRNA profile of purified liver perfusion exosomes (n=5), from patients with uveal melanoma, and five cell lines used as controls, were analysed for the presence of a panel of 88 cancer-related miRNA using RT2 miRNA PCR. The table shows all miRNA that passed quality control; Samples with a Ct value over 35, and samples showing more than one melt peak, with a ratio less than 80% between the major peak and the secondary peak, were excluded from the analysis. As no reference genes are available, miRNA were ranked according to their Ct values for each sample. (PDF 95 KB) [file 12885_2014_5177_MOESM1_ESM.pdf]

Supplementary Table 1. MiRNA profile of purified liver perfusion exosomes (n=5), from patients with uveal melanoma, and five cell lines used as controls, were analysed for the presence of a panel of 88 cancer-related miRNA using RT2 miRNA PCR. The table shows all miRNA that passed quality control; Samples with a Ct value over 35, and samples showing more than one melt peak, with a ratio less than 80% between the major peak and the secondary peak, were excluded from the analysis. As no reference genes are available, miRNA were ranked according to their Ct values for each sample.

Patient 1	Patient 5	Patient 7	Patient 8	Patient 9	A375	MML-1	HTB-133	HTB-177	HMC-1
hsa-miR-9*	hsa-miR-125a-5p	hsa-miR-9*	hsa-miR-9*	hsa-miR-9*	hsa-miR-9*	hsa-miR-9*	hsa-miR-16	hsa-miR-21	hsa-miR-10b
hsa-miR-125a-5p	hsa-miR-9*	hsa-miR-125a-5p	hsa-miR-125a-5p	hsa-miR-129-5p	hsa-miR-125a-5p	hsa-miR-125a-5p	hsa-miR-21	hsa-miR-16	hsa-miR-16
hsa-miR-181b	hsa-miR-216a	hsa-miR-216a	hsa-miR-125b	hsa-miR-107	hsa-miR-181b	hsa-miR-216a	hsa-miR-9*	hsa-miR-25	hsa-miR-9*
hsa-miR-25	hsa-miR-125b	hsa-miR-125b	hsa-miR-216a	hsa-miR-125b	hsa-miR-16	hsa-miR-23a	hsa-miR-15b	hsa-miR-181b	hsa-miR-21
hsa-miR-216a	hsa-miR-9	hsa-miR-25	hsa-miR-107	hsa-miR-125a-5p	hsa-miR-10b	hsa-miR-25	hsa-miR-181b	hsa-miR-149	hsa-miR-146b-5p
hsa-miR-320a	hsa-miR-217	hsa-miR-129-5p	hsa-miR-9	hsa-miR-370	hsa-miR-125b	hsa-miR-125b	hsa-miR-10b	hsa-miR-125b	hsa-miR-25
hsa-miR-217	hsa-miR-320a	hsa-miR-320a	hsa-miR-25	hsa-miR-25	hsa-miR-320a	hsa-miR-217	hsa-miR-191	hsa-miR-15b	hsa-miR-125a-5p
hsa-miR-335	hsa-miR-107	hsa-miR-107	hsa-miR-217	hsa-miR-216a	hsa-miR-21	hsa-miR-335	hsa-miR-216a	hsa-miR-24	hsa-miR-23a
hsa-miR-125b	hsa-miR-129-5p	hsa-miR-217	hsa-miR-19a	hsa-miR-210	hsa-miR-216a	hsa-miR-19a	hsa-miR-25	hsa-miR-23a	hsa-miR-19a
hsa-miR-370	hsa-miR-335	hsa-miR-335	hsa-miR-129-5p	hsa-miR-217	hsa-miR-23a	hsa-miR-133a	hsa-miR-23a	hsa-miR-27a	hsa-miR-335
hsa-miR-19a	hsa-miR-370	hsa-miR-9	hsa-miR-320a	hsa-miR-335	hsa-miR-25	hsa-miR-129-5p	hsa-miR-24	hsa-miR-191	hsa-miR-148a
hsa-miR-133a	hsa-miR-19a	hsa-miR-19a	hsa-miR-335		hsa-miR-217	hsa-miR-21	hsa-miR-148a	hsa-miR-17	hsa-miR-20a
hsa-miR-107	hsa-miR-19b	hsa-miR-181b	hsa-miR-370		hsa-miR-146a	hsa-miR-10b	hsa-miR-125a-5p	hsa-miR-93	hsa-miR-125b
hsa-miR-222*	hsa-miR-181b	hsa-miR-370	hsa-miR-210		hsa-miR-191	hsa-miR-203	hsa-miR-425	hsa-miR-125a-5p	hsa-miR-101
hsa-miR-486-5p	hsa-miR-505	hsa-miR-133a	hsa-miR-323-5p		hsa-miR-133a	hsa-miR-26a	hsa-miR-203	hsa-miR-106a	hsa-miR-182
hsa-miR-425	hsa-miR-25	hsa-miR-19b	hsa-miR-19b		hsa-miR-335	hsa-miR-9	hsa-miR-19b	hsa-miR-9*	hsa-miR-9
hsa-miR-17	hsa-miR-26a	hsa-miR-210	hsa-miR-181b		hsa-miR-486-5p	hsa-miR-183	hsa-miR-26a	hsa-miR-216a	
hsa-miR-19b	hsa-miR-210	hsa-miR-486-5p	hsa-miR-222*		hsa-miR-149		hsa-miR-23b	hsa-miR-20a	
hsa-miR-323-5p	hsa-miR-124	hsa-miR-150			hsa-miR-27a		hsa-miR-30b	hsa-miR-19a	
hsa-miR-200a	hsa-miR-182	hsa-miR-222*			hsa-miR-222*		hsa-miR-19a	hsa-miR-19b	
hsa-miR-324-5p	hsa-miR-323-5p	hsa-miR-200a			hsa-miR-323-5p		hsa-miR-30c	hsa-miR-23b	
hsa-miR-149	hsa-miR-127-5p	hsa-miR-16			hsa-miR-19a		hsa-miR-217	hsa-miR-30c	
hsa-miR-133b	hsa-miR-200a	hsa-miR-323-5p			hsa-miR-9		hsa-miR-20a	hsa-miR-30b	
hsa-miR-182	hsa-miR-149				hsa-miR-324-5p		hsa-miR-27a	hsa-miR-451	
hsa-miR-210	hsa-miR-324-5p				hsa-miR-221		hsa-miR-129-5p	hsa-miR-486-5p	
hsa-miR-130b	hsa-miR-222*				hsa-miR-107		hsa-miR-222*	hsa-miR-10b	
hsa-miR-124	hsa-miR-146b-5p				hsa-miR-19b		hsa-miR-125b	hsa-miR-150	
hsa-miR-505	hsa-miR-21				hsa-miR-200a		hsa-miR-335	hsa-miR-148a	
hsa-miR-296-5p					hsa-miR-124		hsa-miR-150	hsa-miR-425	
hsa-miR-101					hsa-miR-23b		hsa-miR-182	hsa-miR-106b	
hsa-miR-191					hsa-miR-296-5p		hsa-miR-101	hsa-miR-222*	
hsa-miR-30c					hsa-miR-30c		hsa-miR-200a	hsa-miR-29a	
hsa-miR-15a					hsa-miR-129-5p		hsa-miR-451	hsa-let-7b	
hsa-miR-146b-5p					hsa-miR-24		hsa-miR-9	hsa-miR-133a	
hsa-miR-141					hsa-miR-15a			hsa-miR-217	
hsa-miR-218					hsa-miR-26a			hsa-miR-323-5p	
hsa-miR-128					hsa-miR-328			hsa-miR-9	
hsa-miR-106a					hsa-miR-150			hsa-miR-182	
hsa-miR-328					hsa-miR-331-5p			hsa-miR-101	
hsa-miR-23a					hsa-miR-101			hsa-miR-18a	
hsa-miR-129-5p					hsa-miR-29b			hsa-miR-15a	
hsa-miR-106b					hsa-miR-182			hsa-miR-505	
hsa-miR-30b					hsa-miR-15b			hsa-miR-331-5p	
hsa-miR-20a					hsa-miR-218			hsa-miR-296-5p	
hsa-miR-17*					hsa-miR-130b			hsa-miR-335	
hsa-miR-148a					hsa-miR-133b			hsa-miR-31	
hsa-miR-203					hsa-miR-20a			hsa-miR-107	
hsa-miR-130a					hsa-miR-141			hsa-miR-7	
hsa-miR-23b					hsa-miR-106b			hsa-miR-328	
hsa-miR-16					hsa-miR-146b-5p			hsa-miR-128	
hsa-miR-15b					hsa-miR-128			hsa-miR-326	
hsa-miR-150					hsa-miR-148a			hsa-miR-200a	
hsa-miR-127-5p					hsa-miR-7			hsa-miR-203	
hsa-miR-181a					hsa-miR-29a			hsa-miR-129-5p	
hsa-miR-146a					hsa-miR-203			hsa-miR-183	
hsa-miR-24					hsa-miR-127-5p			hsa-miR-127-5p	
hsa-miR-29b					hsa-miR-190			hsa-miR-144	
hsa-miR-190									
