# Supplementary material for: MicroRNA in exosomes isolated directly from the liver circulation in patients with metastatic uveal melanoma
Source: BMC Cancer. 2014 Dec 16;14:962. doi: 10.1186/1471-2407-14-962 (PMC4320618; doi:10.1186/1471-2407-14-962)
Supplement: Supplementary file 2 — Additional file 2: Table S2: Enriched KEGG pathways of predicted targets for the four miRNAs (miR-216a, miR217, miR129-5p and miR-203) in cluster 1. (PDF 26 KB) [file 12885_2014_5177_MOESM2_ESM.pdf]

Supplementary Table 2. Enriched KEGG pathways of predicted targets for the four miRNAs (miR-216a, miR217, miR129-5p and miR-203) in cluster 1.

KEGG pathway	P value	Number of genes	Genes
Glycosaminoglycan biosynthesis - heparan sulfate	4.6E-13	6	EXT1, EXTL2, HS2ST1, HS3ST3B1, NDST3, XYLT1
TGF-beta signaling pathway	1.3E-04	20	ACVR1, ACVR2A, ACVR2B, BMPR2, CREBBP, CUL1, EP300, ID4, MAPK1, RBL2, ROCK1, RPS6KB1, SMAD2. SMAD5, SMAD7, SMAD9, SP1, TGFBR2, XPNPEP3, ZFYVE16
Adipocytokine signaling pathway	1.4E-04	19	ACSL3, G6PC, IKBKB, IRS1, IRS2, JAK2, LEPR, MAPK8, MAPK9, NFKBIA, PPARA, PPARGC1A, PRKAA1, PRKAA2, PRKAB1, PRKAB2, PRKAG2, PTPN11, SOCS3
Fatty acid metabolism	3.4E-04	6	ACADSB, ACOX1, ACSL3, ADH1B, EHHADH, HADHB
ErbB signaling pathway	5.7E-04	24	ABL2, BTC, CAMK2B, ERBB4, JUN, KRAS, MAP2K4, MAPK1, MAPK8, MAPK9, NRG2, NRG3, NRG4, PAK2, PAK7, PIK3CA, PIK3R1, PRKCB, RPS6KB1, SHC4, SOS1, SOS2, SRC, STAT5B
Neurotrophin signaling pathway	5.7E-04	32	BDNF, CALM1, CAMK2B, CAMK4, FOXO3, IKBKB, IRS1, IRS2, JUN, KRAS, MAP3K1, MAP3K5, MAPK1, MAPK8, MAPK9, NFKBIA, NTRK2, NTRK3, PIK3CA, PIK3R1, PTPN11, RAP1A, RAP1B, RAPGEF1, RPS6KA5, SHC4, ,OS1, SOS2, YWHAB, YWHAG, YWHAQ, ZNF274
Wnt signaling pathway	8.5E-04	34	APC, CACYBP, CAMK2B, CCND1, CREBBP, CSNK1A1L, CTBP2, CUL1, CXXC4, DAAM1, DKK1, DKK2, EP300, FZD4, FZD6, JUN, MAP3K7, MAPK8, MAPK9, NFAT5, NLK, PPP3CA, PRICKLE2, PRKCB, ROCK1, SIAH1, SMAD2, BL1X, TCF7, TCF7L2, VANGL2, WNT3, WNT5A, XPNPEP3
Focal adhesion	8.5E-04	43	ACTN1, ACTN4, ARHGAP5, CAV2, CCND1, COL1A1, COL4A1, COL4A4, COL5A1, FN1, HGF, IGF1, IGF1R, ITGA1, ITGA2, ITGA6, ITGB6, JUN, LAMA4, MAPK1, MAPK8, MAPK9, MYLK3, PAK2, PAK7, PDGFD, PIK3CA, IK3R1, PPP1R12A, PRKCB, PXN, RAP1A, RAP1B, RAPGEF1, ROCK1, SHC4, SOS1, SOS2, SRC, TLN1, TNC, VAV3, XIAP
Glioma	1.4E-03	15	CALM1, CAMK2B, CCND1, CDK6, E2F3, IGF1, IGF1R, KRAS, MAPK1, PIK3CA, PIK3R1, PRKCB, SHC4, SOS1, SOS2
Pathways in cancer	1.4E-03	59	APC, APPL, ARNT, BRCA2, CCDC6, CCND1, CDK6, COL4A1, COL4A4, CREBBP, CTBP2, E2F3, EP300, ETS1, EVI1, FGF13, FGF2, FGFR2, FN1, FZD4, FZD6, GLI3, HDAC2, HGF, IGF1, IGF1R, IKBKB, ITGA2, ITGA6, JUN, KITLG, RAS, LAMA4, MAPK1, MAPK8, MAPK9, NFKBIA, PIK3CA, PIK3R1, PLD1, PRKCB, PTCH1, RALGDS, RASSF5, RUNX1, RUNX1T1, SMAD2, SOS1, SOS2, STAT5B, TCF7, TCF7L2, TGFBR2, TPM3, TPR, WNT3, WNT5A, XIAP, XPNPEP3
Type II diabetes mellitus	3.4E-03	13	CACNA1A, CACNA1B, CACNA1C, IKBKB, IRS1, IRS2, MAPK1, MAPK8, MAPK9, PIK3CA, PIK3R1, PRKCE, SOCS3
Taurine and hypotaurine metabolism	3.8E-03	4	C10orf22, CSAD, GAD1, GAD2
Renal cell carcinoma	5.3E-03	19	ARNT, CREBBP, EP300, ETS1, HGF, JUN, KRAS, MAPK1, PAK2, PAK7, PIK3CA, PIK3R1, PTPN11, RAP1A, RAP1B, RAPGEF1, SOS1, SOS2, XPNPEP3

ECM-receptor interaction	8.0E-03	13	CD47, COL1A1, COL4A1, COL4A4, COL5A1, FN1, ITGA1, ITGA2, ITGA6, ITGB6, LAMA4, SV2B, TNC
Chronic myeloid leukemia	8.3E-03	19	CCND1, CDK6, CTBP2, E2F3, EVI1, HDAC2, IKBKB, KRAS, MAPK1, NFKBIA, PIK3CA, PIK3R1, PTPN11, RUNX1, SHC4, SOS1, SOS2, STAT5B, TGFBR2
Colorectal cancer	1.5E-02	15	APC, APPL, CCND1, JUN, KRAS, MAPK1, MAPK8, MAPK9, PIK3CA, PIK3R1, RALGDS, SMAD2, TCF7, TCF7L2, TGFBR2
Non-small cell lung cancer	1.6E-02	12	CCND1, CDK6, E2F3, FOXO3, KRAS, MAPK1, PIK3CA, PIK3R1, PRKCB, RASSF5, SOS1, SOS2
Acute myeloid leukemia	2.0E-02	14	CCND1, IKBKB, KRAS, MAPK1, PIK3CA, PIK3R1, RPS6KB1, RUNX1, RUNX1T1, SOS1, SOS2, STAT5B, TCF7, TCF7L2
Aldosterone-regulated sodium reabsorption	2.1E-02	12	ATP1B1, ATP1B3, IGF1, IRS1, IRS2, KRAS, MAPK1, NEDD4L, NR3C2, PIK3CA, PIK3R1, PRKCB
GnRH signaling pathway	2.3E-02	21	ADCY2, CACNA1C, CALM1, CAMK2B, GNAS, GNRHR, ITPR1, ITPR2, JUN, KRAS, MAP2K4, MAP3K1, MAP3K2, MAPK1, MAPK8, MAPK9, PLD1, PRKCB, SOS1, SOS2, SRC
B cell receptor signaling pathway	2.5E-02	17	BCL10, CR2, DAPP1, IKBKB, JUN, KRAS, LILRA6, MAPK1, NFAT5, NFKBIA, PIK3CA, PIK3R1, PPP3CA, PRKCB, SOS1, SOS2, VAV3
Prostate cancer	3.0E-02	20	CCND1, CREB3L2, CREB5, CREBBP, E2F3, EP300, FGFR2, IGF1, IGF1R, IKBKB, KRAS, MAPK1, NFKBIA, PDGFD, PIK3CA, PIK3R1, SOS1, SOS2, TCF7, TCF7L2
Insulin signaling pathway	3.1E-02	27	CALM1, EIF4E2, G6PC, IKBKB, IRS1, IRS2, KRAS, MAPK1, MAPK8, MAPK9, PIK3CA, PIK3R1, PPARGC1A, PPP1R3D, PRKAA1, PRKAA2, PRKAB1, PRKAB2, PRKAG2, RAPGEF1, RHOQ, RPS6KB1, SHC4, SOCS3, SORBS1, SOS1, SOS2
mRNA surveillance pathway	3.5E-02	15	CPSF2, CSTF2T, ETF1, HBS1L, NUDT21, NXT2, PABPC1L, PABPC1L2A, PABPC3, PABPC5, PABPCP5, PABPN1, PAPOLA, PPP2R3A, UPF2
Arrhythmogenic right ventricular cardiomyopathy (ARVC)	3.7E-02	16	ACTN1, ACTN4, CACNA1C, CACNB4, CACNG2, CDH2, DMD, ITGA1, ITGA2, ITGA6, ITGB6, PKP2, RYR2, SGCD, TCF7, TCF7L2
Long-term potentiation	3.7E-02	16	CACNA1C, CALM1, CAMK2B, CAMK4, CREBBP, EP300, GRIN2A, ITPR1, ITPR2, KRAS, MAPK1, PPP1R12A, PPP3CA, PRKCB, RAP1A, RAP1B
Adherens junction	3.8E-02	18	ACTN1, ACTN4, CREBBP, EP300, IGF1R, LMO7, MAP3K7, MAPK1, NLK, PTPRB, PVRL3, SMAD2, SNAI2, SORBS1, SRC, TCF7, TCF7L2, TGFBR2
Endocytosis	4.7E-02	33	ACAP2, AP2B1, ASAP1, ASAP2, CAV2, CCR5, CHMP2B, CLTC, DAB2, DNAJC6, DNM3, EEA1, ERBB4, FGFR2, IGF1R, IQSEC3, NEDD4L, PDCD6IP, PLD1, PSD3, RAB11A, RAB22A, RAB4A, SH3GLB1, SH3KBP1, SMAD2, SMAD7, SRC, TGFBR2, USP8, VPS24, ZFYVE16, ZFYVE20
