# Supplementary material for: MicroRNA in exosomes isolated directly from the liver circulation in patients with metastatic uveal melanoma
Source: BMC Cancer. 2014 Dec 16;14:962. doi: 10.1186/1471-2407-14-962 (PMC4320618; doi:10.1186/1471-2407-14-962)
Supplement: Supplementary file 3 — Additional file 3: Table S3: Enriched KEGG pathways of predicted targets for the seven miRNAs (miR-9*, miR125a-5p, miR-25, miR-125b, miR-335, miR-19a and miR-9) in cluster 2. (PDF 17 KB) [file 12885_2014_5177_MOESM3_ESM.pdf]

Supplementary Table 3. Enriched KEGG pathways of predicted targets for the seven miRNAs (miR-9*, miR125a-5p, miR-25, miR-125b, miR-335, miR-19a and miR-9) in cluster 2.

KEGG pathway	P value	Number of genes	Genes
Glycosaminoglycan biosynthesis - heparan sulfate	3.98E-07	5	EXT1, GLCE, HS3ST3B1, HS6ST1, HS6ST2
Mucin type O-Glycan biosynthesis	7.03E-06	6	GALNT12, GALNT13, GALNT14, GALNT3, GALNTL1, GCNT1
Biotin metabolism	1.38E-05	1	HLCS
Pantothenate and CoA biosynthesis	2.19E-03	7	BCAT1, BCAT2, ENPP1, PANK1, PANK3, PPCS, VNN1
Glioma	2.19E-03	15	CALM1, CALM2, CDK6, E2F2, IGF1, PDGFRA, PDGFRB, PIK3CA, PIK3R1, PIK3R3, PLCG1, PTEN, RB1, SHC1, SHC2
N-Glycan biosynthesis	1.40E-02	8	ALG1, MAN1A1, MAN1A2, MAN1B1, MGAT4A, MGAT5, ST6GAL1, STT3B
Endocytosis	1.40E-02	35	ACAP2, ARAP2, ARF6, ASAP2, CAV2, CBL, CCR5, CHMP2B, CXCR4, EHD4, GIT2, ITCH, LDLRAP1, NEDD4, PARD6B, PDCD6IP, PDGFRA, PLD1, PSD3, RAB11A, RAB11FIP4, SH3GLB1, SH3KBP1, SMAD2, SMAP2, SMURF1, SMURF2, STAM, TGFBR2, TRAF6, USP8, VPS37C, VPS4B, WWP1, ZFYVE16
Cysteine and methionine metabolism	1.57E-02	9	DNMT3A, GOT1, GOT2, LDHB, MAT1A, MAT2B, MTAP, MTR, TRDMT1
Lysine degradation	1.59E-02	10	DLSTP, EHHADH, GLT25D2, MLL, MLL3, SETD2, SETD7, SUV39H1, SUV420H2, TMLHE
Arrhythmogenic right ventricular cardiomyopathy (ARVC)	1.57E-02	16	ACTN2, CACNB1, CACNB2, CACNB3, CTNNA1, DMD, ITGA2, ITGA6, ITGB1, ITGB4, JUP, LMNA, RYR2, SGCD, SLC8A1, TCF7
Axon guidance	1.73E-02	27	ABL1, ABLIM1, ABLIM3, CFL2, CXCR4, EFNA1, EPHA4, EPHA7, EPHB4, GNAI1, ITGB1, NFAT5, NFATC1, NFATC2, NRP1, PAK2, RASA1, ROBO1, ROCK1, SEMA4C, SEMA4D, SEMA4F, SEMA5A, SEMA6A, SRGAP3, UNC5C, UNC5D
Protein processing in endoplasmic reticulum	2.39E-02	29	AC079305.6-2, ATXN3, ATXN3L, BAK1, DERL1, DNAJA1, DNAJC3, EIF2AK2, EIF2AK3, EIF2S1, HERPUD1, HSPH1, LMAN1, MAN1A1, MAN1A2, MAN1B1, MAP2K7, MAPK10, MARCH6, SEL1L, SSR1, SSR3, STT3B, SYVN1, TRAM1, UBE2D3, UBE2G1, UBE4B, YOD1
Insulin signaling pathway	4.32E-02	27	ACACB, CALM1, CALM2, CBL, EIF4E, FLOT2, FOXO1A, HK2, INPP5D, MAPK10, PDPK1, PHKA1, PIK3CA, PIK3R1, PIK3R3, PPARGC1A, PPP1CB, PPP1R3A, PRKAA2, PRKAG2, PRKAR1A, RHOQ, SHC1, SHC2, SOCS1, SOCS4, TSC1
